# Hay Fever Hints

**Published:** 1912-09

**Authors:** 


Hay Fever Hints.—We are now well into the season when the services of the physician are urgently demanded by the victim of vasomotor rhinitis—a season dreaded not alone by the patient, but, not uncommonly, by his medical adviser as well. Particularly is this true of the latter if he has not kept abreast of the most modern ideas on the therapy of hay fever. In any event the disease is one that tries the patience and calls for the application of remedial agents that have been proved beyond peradventure.

In the treatment of hay fever the physician rarely has an opportunity for the application of preventive measures. His help is usually sought only after the attack has manifested itself—when the patient is suffering (acutely in most cases) from the ravages of the disease. Effective treatment is then demanded—and promptly, too. Administration of the suprarenal substance in the form of its isolated active principle, Adrenalin, is undoubtedly the wise procedure at this juncture. One feels safe in saying this in view of the long and effective service which has been rendered by this agent in critical emergencies.

There are a number of forms in which Adrenalin is successfully used in the treatment of hay fever. Adrenalin Chloride Solution and Adrenalin Inhalant come naturally to mind in this connection. The substance is also incorporated in the several Anesthone preparations—in Anesthone Cream, Anesthone Inhalant, and Anesthone Tape, all worthy of confidence, and especially worthy of trial in cases in which for any reason the older Andrenalin products seem not to be indicated. The Andrenalin and Anesthone products, as is well known perhaps to most physicians, are manufactured by Parke, Davis & Co. An exposition of their uses in the malady in question, together with the technique of administration, is now appearing in the commercial pages of the leading medical publications. Practitioners are advised to consult these current announcements.

